# Examining the effectiveness of ChatGPT responses to frequently asked questions by individuals with postural disorders

**DOI:** 10.1590/1806-9282.20250750

**Published:** 2025-12-15

**Authors:** Burcu Dursun, Mustafa Savaş Torlak, Osman Tufekci

**Affiliations:** 1 KTO Karatay University, Vocational School of Health Sciences, Department of Therapy and Rehabilitation – Konya, Türkiye.; 2 Farabi Hospital, Department of Physical Therapy – Konya/Türkiye.

**Keywords:** Artificial intelligence, Physical therapy, Posture

## Abstract

**OBJECTIVE:**

The aim of the study was to evaluate the quality and readability of ChatGPT responses to frequently asked questions by individuals with posture disorder. Providing reliable and evidence-based information about posture disorders is vital for individuals to be correctly informed.

**METHODS:**

The 10 most frequently asked questions about posture disorder were selected by two researchers from a list created by ChatGPT. The questions were transmitted to ChatGPT 4.0, and the initial responses were recorded without further follow-up questions. The quality of the responses was then assessed by five independent experts (three physiotherapists, one physical therapy and rehabilitation specialist, and one orthopedics and traumatology specialist) with a four-grade evaluation system. Readability levels were analyzed with the Flesch-Kincaid Grade Level through WordCalc software. Statistical analysis was performed using Statistical Package for the Social Sciences v29.0, and intraclass correlation coefficients were used to measure inter-rater reliability.

**RESULTS:**

Following a thorough evaluation of the 10 responses received, six were rated as "Excellent responses requiring no explanation," while a further four were designated as "Satisfactory responses requiring minimal explanation." The median quality score of the responses was high, indicating good alignment with current evidence-based practice. The average readability level of the responses was determined to be 8.4. Inter-rater reliability was good, with an intraclass correlation coefficients value of 0.756.

**CONCLUSION:**

ChatGPT provides relatively coherent and generally readable answers to frequently asked questions about posture disorders, with most needing minimal explanation. While promising as a resource to meet the information needs of people with posture disorders, further improvements are needed to align it with personalized health needs.

## INTRODUCTION

Recent years have witnessed significant advancements in the field of artificial intelligence (AI) technology. AI-supported chatbots are computer programs capable of understanding human language and generating appropriate responses, and can interact with users in depth^1^. These programs are trained with deep learning algorithms over large amounts of text data from the internet, enabling them to develop an understanding of and respond to a wide range of topics^2^. ChatGPT, an AI chatbot developed by OpenAI, employs deep learning technology to provide human-like responses to natural language inputs^3^. ChatGPT has attracted attention for its user-friendly interface and has the potential to create substantial impacts on healthcare services and patient education^4^.

Posture is defined as the position of the body when sitting or standing^5^. Variations in posture may be attributable to age, gender, body development, and environmental or psychological factors^6^. Postural disorder (PD) is a multifactorial condition that arises from muscle imbalances, nutritional issues, inactivity, poor posture habits, improper bag carrying, and psychological factors^7^. PD can lead to muscle weakness, resulting in aesthetic problems, pain, and musculoskeletal diseases^8^. Additionally, PD is a prevalent yet often disregarded health concern on a global scale^9^. Therefore, individuals affected by PDs may turn to AI systems like ChatGPT for the procurement of information.

Despite its current limitations, ChatGPT demonstrates considerable potential in filtering scientific content and contributing to the development of personalized treatment processes^10^. A recent study^11^ drew parallels between ChatGPT's recommendations for various musculoskeletal disorders and the APTA Academy of Orthopedic Physical Therapy's Clinical Practice Guidelines (CPGs), finding that ChatGPT exhibited high levels of accuracy and reliability. Researchers have indicated that ChatGPT has potential as a clinical support tool in rehabilitation. Furthermore, several other studies evaluated ChatGPT's responses to the most frequently asked questions from patients about hip arthroscopy^12^, ankle arthroplasty^13^, and total shoulder arthroplasty^14^. In these studies, ChatGPT's responses to the aforementioned questions were found to be generally satisfactory and adequate, with minimal additional clarification required in most cases.

The quality of the responses provided by ChatGPT to questions regarding PD remains to be ascertained. The objective of this study was to evaluate the quality and readability of the responses provided by ChatGPT to inquiries concerning PD.

## METHODS

### Question collection process

A prompt was entered into the ChatGPT application as "What are the 50 most frequently asked questions by those with PDs?" Following a thorough examination of the pool of questions, a total of 10 questions were selected by two researchers (two physiotherapists) and can be seen in Table 1.

**Table 1:** Questions.

Question number	Question
1	Which doctor should I see to treat a posture disorder?
2	How do I know whether I have a posture disorder?
3	I'm constantly hunched over; how can I fix it?
4	If I use the wrong pillow and mattress, will my posture be damaged?
5	Which exercises can I do to improve my posture?
6	How long does it take to correct my posture?
7	Should I use a posture-correcting corset?
8	Can posture disorders that occur in childhood be corrected in adulthood?
9	Does excess weight impair my posture?
10	Does carrying a bag on one shoulder ruin my posture?

### ChatGPT usage

The questions were formulated using ChatGPT 4.0. In order to prevent ChatGPT from being influenced by prior interactions, the browser history was initially erased, and then a new ChatGPT account was established for the initial use. In the subsequent phase, 10 questions determined by the researchers were asked in order, and the initial responses of ChatGPT to each question were meticulously recorded. At this time, no further questions were posed, and no additional explanations were made.

### Evaluation

The quality of the responses was assessed by five independent researchers (three physiotherapists, one physical therapy and rehabilitation specialist, and one orthopedics and traumatology specialist) using the four-grade system proposed by Mika et al.^15^:

1. "Excellent response, no explanation needed": The answer was considered extremely accurate and comprehensive, thus providing information without requiring any additional explanation.
2. "Satisfactory, requires minimal explanation": The answer was rated correct but required minimal supplementary explanation to fully address the user's question.
3. "Satisfactory, needs moderate explanation": Although the answer was correct, moderate additional explanation was necessary to meet the user's needs.
4. "Unsatisfactory, requires significant explanation": The response contains significant misinformation or is considered overly generalized, which may lead to user misunderstanding.

The readability of ChatGPT responses was assessed by means of WordCalc software, whereby responses to each question were pasted into a readability calculator, and the corresponding Flesch-Kincaid Rating Level was recorded.

### Statistics

The data evaluation process involved the utilization of the SPSS (Statistical Package for the Social Sciences) Statistics for Windows, Version 29.0 (IBM Corp., Armonk, NY, USA) software package. The results of the quality assessment of the responses are given as median (min–max). The inter-rater reliability was calculated using intraclass correlation coefficients (ICC). The ICC values were considered and then categorized as poor for <0.50, moderate for 0.50–0.75, good for 0.75–0.90, and excellent for >0.90^16^.

## RESULTS

The study was conducted with ChatGPT version 4.0 in February 2025. Six of the 10 responses evaluated in the study were evaluated and classified as "Excellent response, no explanation needed," while four were designated as "Satisfactory, requires minimal explanation." In addition to the median scores of the responses, the average readability level was determined to be 8.4, as shown in Table 2. Differences among expert evaluations were observed for certain questions, reflecting a diversity of opinions among the raters; this is illustrated in Table 3.

**Table 2:** Readability and quality of responses.

Question number	Flesch-Kincaid grade level	Response quality		
		Median	Min–Max	ICC
1	12	1	1–3	0.756
2	7	1	1–2	
3	6.3	2	1–2	
4	6.7	1	1–1	
5	5.4	2	1–2	
6	6.7	2	1–3	
7	8.3	1	1–1	
8	12	2	1–2	
9	10.2	1	1–2	
10	9.4	1	1–2	

ICC: intraclass correlation coefficients.

**Table 3:** Diversity of expert opinions.

Question	Physiotherapist 1	Physiotherapist 2	Physiotherapist 3	Physical therapy and rehabilitation specialist	Orthopedics and traumatology specialist
1	2	1	1	1	3
2	2	1	1	1	2
3	2	1	2	1	2
4	1	1	1	1	1
5	2	2	1	2	2
6	2	3	1	2	3
7	1	1	1	1	1
8	1	2	2	1	2
9	2	1	1	1	2
10	2	1	1	1	1

Question 1: The question "Which doctor should I see to treat a posture disorder?" was evaluated as "Excellent response, no explanation needed" by three evaluators, as "Satisfactory, requires minimal explanation" by one evaluator, and as "Satisfactory, needs moderate explanation" by one evaluator, and the readability level was determined as 12.

Question 2: The question "How do I know whether I have a posture disorder?" was evaluated as "Excellent response, no explanation needed" by three evaluators and as "Satisfactory, requires minimal explanation" by two evaluators, and the readability level was calculated as seven.

Question 3: The question "I'm constantly hunched over; how can I fix it?" was evaluated by two evaluators as "Excellent response, no explanation needed" and by three evaluators as "Satisfactory, requires minimal explanation," and the readability level was ascertained as 6.3.

Question 4: The question "If I use the wrong pillow and mattress, will my posture be damaged?" was assessed by all evaluators as "Excellent response, no explanation needed," and the readability level was established as 6.7.

Question 5: For the question "Which exercises can I do to improve my posture?," the readability level was determined to be 5.4, with one assessor rating the answer as "Excellent response, no explanation needed" and four assessors rating the answer as "Satisfactory, requires minimal explanation."

Question 6: The readability level of the question "How long does it take to correct my posture?" was 6.7, and one assessor rated the answer as "Excellent response, no explanation needed," two assessors rated it as "Satisfactory, requires minimal explanation," and two assessors rated it as "Satisfactory, needs moderate explanation."

Question 7: The question "Should I use a posture correcting corset?" was evaluated as "Excellent response, no explanation needed" by all evaluators, and the readability level was determined as 8.3.

Question 8: The question "Can posture disorders that occur in childhood be corrected in adulthood?" was evaluated by two evaluators as "Excellent response, no explanation needed" and three evaluators as "Satisfactory, requires minimal explanation," and the readability level was established as 12.

Question 9: The question "Does excess weight impair my posture?" was evaluated as "Excellent response, no explanation needed" by three assessors and as "Satisfactory, requires minimal explanation" by two assessors, and the readability level was found to be 10.2.

Question 10: The readability level of the question "Does carrying a bag on one shoulder ruin my posture?" scored 9.4, with four assessors rating the answer as "Excellent response, no explanation needed" and one assessor rating it as "Satisfactory, requires minimal explanation."

## DISCUSSION

In this study, the quality and readability of ChatGPT 4.0 responses related to PD were evaluated. ChatGPT provides answers to frequently asked questions about PDs that are partially consistent with expert opinions, but further verification is required to confirm complete clinical accuracy. In terms of readability, the responses were found to be above the recommended sixth-grade level for patient education materials. The average Flesch-Kincaid reading level was calculated as 8.4, which is above the recommended level for general patient-focused content. This suggests that ChatGPT responses may negatively impact their understandability, particularly for individuals with limited health literacy.

This study is pioneering, namely the first study, in its evaluation of ChatGPT responses on PDs. In the physiotherapy and musculoskeletal system, the accuracy and effectiveness of ChatGPT have been examined through various studies and methodologies, yielding different results. One recent study found that ChatGPT answered clinical questions related to the musculoskeletal system with 80% accuracy, and its responses were consistent in terms of reliability^11^. These findings serve to reinforce the potential of ChatGPT to function as a reliable resource within the domain of clinical decision support systems. AlShehri et al. assessed the responses to common patient questions about hip arthroscopy and noted that ChatGPT 3.5 provided satisfactory findings in terms of accuracy and completeness, though some errors should be considered before using it for patient education^17^. Similarly, the research by Artioli et al. found that most ChatGPT responses regarding ankle arthroplasty were rated as excellent, although in some instances, more detailed explanations were needed^13^. Conversely, the result of another study revealed that ChatGPT offered effective evidence-based answers to frequently asked patient questions in evaluations regarding total hip arthroplasty^15^. Assessments of the accuracy and consistency of ChatGPT's responses to inquiries related to total knee arthroplasty^18,19^ indicate that this system holds significant potential as a patient education and clinical decision support tool.

Our study and similar studies in the literature demonstrate the potential of ChatGPT to provide accurate and effective information to patients regarding the musculoskeletal system. However, in some cases, more detailed explanations and up-to-date information are required, which may limit the use of ChatGPT as a clinical decision support tool. There is a need for further research into the ability of ChatGPT to provide accurate and reliable information to patients.

In order to enhance the objectivity of ChatGPT's responses and ensure that the evaluations are grounded in evidence-based principles, an additional comparative analysis was conducted. Within this scope, the responses provided by ChatGPT to the selected questions were compared with peer-reviewed academic sources. Table 4 presents this comparison, highlighting both areas where the responses closely align with scientific literature and instances where content limitations were identified. This approach was adopted to reduce the subjectivity inherent in expert-based evaluations and to enable a more standardized and reliable framework for assessing the accuracy of AI-generated health information.

**Table 4:** Comparison of ChatGPT responses with sources.

Question	Artificial intelligence	References	Consistency
1. Which doctor should I see to treat a posture disorder?	Support should be sought from a physical medicine and rehabilitation (PM&R) specialist, an orthopedic specialist, and a physiotherapist. PM&R consultation is recommended in cases of pain, orthopedic specialists for structural problems, and physiotherapists for mild functional impairments.	A multidisciplinary and disciplinary team approach is essential in the prevention, diagnosis, and treatment of postural disorders. Physiotherapists, physical medicine and rehabilitation (PM&R) specialists, and, when necessary, orthopedic specialists should work collaboratively^20^.	High consistency: ChatGPT's suggestions mostly align with the reference statement.
2. The question "How do I know whether I have a posture disorder?	It recommends home-applicable tests (mirror test, wall test, and photograph) and symptom-based self-assessment	A detailed postural analysis using clinical, multidisciplinary, and technological methods is recommended^20^.	Moderate consistency: The core ideas are similar, but the depth and methodology differ.
3. The question "I'm constantly hunched over; how can I fix it?	Exercises to enhance postural awareness and strengthen the back, shoulder, and neck muscles aimed at correcting kyphotic posture; ergonomic adjustments; and, if necessary, the use of braces and physiotherapy support are recommended.	Treatment of kyphosis includes strengthening, mobility, postural education, balance training, and professional rehabilitation programs tailored to the patient's age and condition; while braces serve as supportive devices, they are not sufficient as a sole intervention^20^.	High consistency: The recommended methods and target muscle groups are similar.
4. The question "If I use the wrong pillow and mattress, will my posture be damaged?	Incorrect pillows and mattresses can disrupt the natural alignment of the spine, potentially leading to neck and back pain. The use of an appropriate pillow (supporting the cervical curve and with suitable height) and a medium-firm mattress is recommended. Sleep position is also important.	An appropriate pillow design, featuring elevated edges for side sleepers and a lower, flattened central area for those sleeping on their backs, promotes optimal spinal alignment and helps alleviate neck pain^21^.	Moderate consistency: While ChatGPT discusses general effects, the study provides scientific evidence demonstrating the benefits of specific applications.
5. For the question "Which exercises can I do to improve my posture?	Exercises that strengthen the back, shoulder, neck, and core muscles are recommended, and the importance of increasing postural awareness in daily habits is emphasized.	It is noted that regular exercise improves posture, especially through strengthening the back and core muscles. Alongside exercise, enhancing postural awareness and making lifestyle changes are emphasized as crucial components^22^.	High consistency: Information consistency and details are highly similar.
6. The readability level of the question "How long does it take to correct my posture?	Noticeable improvement occurs within 2–4 weeks of regular exercise, while complete postural correction takes place between 3 and 6 months.	The most effective outcomes are achieved through exercise programs targeting the nervous and muscular systems, conducted three times weekly for 15–60 min per session, lasting at least 6 weeks^23^.	Moderate consistency: While ChatGPT summarizes the general durations and processes, the scientific study provides detailed information on exercise frequency and duration.
7. The question "Should I use a posture correcting corset?	Braces are temporarily recommended to correct posture and increase awareness. Daily use of 1–2 h is advised, as prolonged use may lead to muscle weakening, and ill-fitting models can cause discomfort. To enhance effectiveness, brace use should be supported by exercise.	The application of braces targets multiple clinical objectives, including alleviating pain, correcting deformities, providing muscular support, and maintaining spinal alignment. Nevertheless, long-term usage has been highlighted to possibly reduce muscle activation and lead to complications affecting the skin and internal organs. Furthermore, issues related to social acceptance and risks associated with the brace's materials and design have been thoroughly investigated^24^.	Moderate consistency: ChatGPT responses do not address social acceptance and clinical considerations, and design features are only superficially discussed.
8. Can posture disorders that occur in childhood be corrected in adulthood?	Muscle weakness and functional impairments related to habitual behaviors can be improved with exercise; however, in advanced structural disorders, exercise may provide benefits, but surgical intervention might be necessary for complete correction.	Rehabilitation can lead to improvement in functional postural disorders in adults. For structural deformities (such as congenital scoliosis and Scheuermann's kyphosis), surgical and multidisciplinary interventions are recommended^20^.	High consistency: There is strong consistency in information and details.
9. Does excess weight impair my posture?	Overweight conditions can place additional load on the lower back, thoracic spine, and joints, potentially causing postural abnormalities such as increased thoracic kyphosis, weakened core muscles, and the formation of lordosis.	In obese individuals, thoracic kyphosis increases, and spinal as well as hip mobility decreases. Weight is a significant factor affecting spinal mechanics and should be considered in rehabilitation programs^25^.	Moderate to high consistency: ChatGPT accurately conveys the general trends; however, it does not address the specific biomechanical aspects of the changes.
10. The readability level of the question "Does carrying a bag on one shoulder ruin my posture?	It has been indicated that carrying a bag on a single shoulder leads to postural asymmetry, muscular imbalance, and associated pain.	Unilateral load carrying leads to postural asymmetry, muscular imbalance, elevated pain levels, and impaired balance^26^.	High consistency: Consistent with the literature and recommendations are parallel.

### Strengths and limitations

This study has several strengths that increase its relevance for assessing the role of ChatGPT in PD. Firstly, this study is the first to assess the quality and accuracy of AI responses in relation to PD. This focus meets a significant knowledge gap by handling the need for accurate and accessible information about PD and related treatment methods.

This study has several limitations. Although this study aimed to identify frequently asked questions (FAQs) about PDs, the selected questions were generated by ChatGPT. This introduces a potential selection bias, as the questions may reflect the assumptions of the AI rather than the actual needs of patients. The answers provided by ChatGPT can sometimes be superficial for complex and multidisciplinary health issues, such as posture problems. Moreover, some of the included questions are inherently ambiguous, and their answers may vary significantly depending on individual factors. Given the complex and personalized nature of PDs, the responses provided by ChatGPT are often limited to generalized information. The model is also prone to generating hallucinations, such as fabricated references or inaccurate clinical suggestions. Furthermore, since ChatGPT has been trained predominantly on data from Western medical perspectives, its responses may lack relevance or sensitivity to different cultural and regional contexts. Especially in situations that require clinical decisions, more in-depth and expert-based information is needed. Additionally, the process of relying on expert judgment to assess the quality of responses can introduce subjectivity, even when standardized criteria are applied. Another limitation is that ChatGPT is not connected to a constantly updated source database. Therefore, there is a risk that future research on PD may not be reflected in the model. It should also be acknowledged that AI models are continuously evolving, and future versions may demonstrate significantly different performance. Therefore, comparative evaluations across different AI versions are essential. In our study, the reading level of the content was found to be above ideal standards. This may pose accessibility challenges for individuals with low health literacy and highlights the importance of optimizing readability in the future development of AI-generated health content. Future research should investigate the accuracy and effectiveness of the information provided by ChatGPT regarding PDs with different user profiles and evaluate the management of these conditions using multidisciplinary approaches.

## CONCLUSION

ChatGPT is a promising complementary tool for providing education and information about PD, but it cannot replace professional health advice. Developments in AI technology and regular refinement in line with updated scientific evidence may increase the reliability and effectiveness of the model. Future research should monitor the quality of the model's responses over time in order to evaluate performance trends such as consistency, improvement, or decline. The impact of the information provided by ChatGPT on clinical outcomes—particularly in terms of patient adherence, comprehension, and behavioral change—should be examined in detail. Moreover, the role of multi-step and interactive dialogues in real-life patient education may constitute a distinct area of investigation. In addition, conducting version-based comparisons among continuously evolving AI models is essential to identify potential differences in performance. Future studies should aim to enhance ChatGPT's capacity to provide information on PDs by developing user-centered FAQ content based on data obtained from clinical interviews, surveys, or digital health platforms. In doing so, the integration of AI-powered tools into clinical practice can be grounded on a more robust foundation.

## Data Availability

The datasets generated and/or analyzed during the current study are available from the corresponding author upon reasonable request.
